# A Path Analysis of Nutrition, Stimulation, and Child Development Among Young Children in Bihar, India

**DOI:** 10.1111/cdev.13057

**Published:** 2018-03-12

**Authors:** Leila M. Larson, Reynaldo Martorell, Patricia J. Bauer

**Affiliations:** ^1^ Emory University

## Abstract

Nutrition plays an important role in the development of a child, particularly in low‐ and middle‐income countries where malnutrition is often widespread. The relation between diet, hemoglobin, nutritional status, motor development, stimulation and mental development was examined in a cross‐sectional sample of 1,079 children 12–18 months of age living in rural Bihar, India. Path analysis revealed associations between (a) length‐for‐age *z*‐scores and motor development, standardized β (β) = .285, *p* < .001, and (b) motor and all mental development outcomes (language: β = .422; personal‐social: β = .490; memory: β = .139; and executive function: β = .072, all *p* < .001). Additionally, stimulation was significantly associated with language scores and hemoglobin concentration with memory. These findings inform interventions aimed at improving child development in Northern India.

Infancy and early childhood are periods of additive neural development (increasing cells, neurons, synapses, etc.) and especially rapid physical and mental development. Poor nutrition during early life can have considerable and long‐lasting consequences on development. The study of the relations between malnutrition and child development is critical to identifying modifiable predictors of development on which to intervene. Many studies have investigated individual associations between diet, nutritional status, stimulation, and mental development (Barros, Matijasevich, Santos, & Halpern, 2010; Grantham‐McGregor et al., 2007; Hadley, Tegegn, Tessema, Asefa, & Galea, 2008; Servili et al., 2010; Sudfeld et al., 2015). Yet only a few have examined the relations simultaneously (Olney et al., 2009, 2013; Pollitt, Jahari, & Walka, 2000; Prado et al., 2017; Walka & Pollitt, 2000). Furthermore, a comprehensive examination of these influences has not been performed for specific cognitive functions. The current analysis adds a unique perspective by investigating a theoretical framework for how nutrition relates to memory and executive function, in addition to language and personal‐social development in young children in rural Bihar, India.

Pollitt (2000) hypothesized that cognitive development is influenced by nutritional status, physical growth, motor development and activity, as well as interactions among them, and between children and their social and physical environments. The study of these relations is important, particularly in low‐ and middle‐income countries (LMICs) where many children suffer from malnutrition, poor diets, and food deprivation. One exemplar finding comes from a study of Indonesian children 12–18 months of age, in which a structural equation model of longitudinal data identified significant direct pathways between energy intake and motor activity, subsequently affecting motor and mental development (Pollitt et al., 2000). In a cross‐sectional study of Tanzanian children 5–19 months of age, length‐for‐age *z*‐score (LAZ) was associated with motor activity. In turn, motor activity was positively associated with language development and object manipulation, and negatively associated with children's tendency to fuss and time being carried (Olney et al., 2009). Associations varied by age such that LAZ and motor development, and motor and language, fussing, and object manipulation were more strongly related in older children, whereas associations between motor and carrying were stronger in younger children (Olney et al., 2009). Psychosocial stimulation provided to the child was not measured in the study, but could play an important mediating role.

Individual associations between predictors and mental development of children are informative. Yet it is important to additionally examine the interplay between predictors and their indirect effects on development. Such examinations may be especially important in at‐risk populations in which it can be expected that the multiple causes of anemia (diet, inflammation, infection, genetics, etc.), nutritional status, and motor and mental development and covariance among predictors increase the complexity of the predictive models. Interventions aimed at improving mental development in malnourished populations can use this information to target particular predictors or pathways and enhance cost‐effective impact. Furthermore, improving more than one predictor may have additive or synergistic effects on development. For example, a study in Pakistan demonstrated an additive effect of the combined delivery of nutrition supplementation and psychosocial stimulation on mental development at 4 years of age (Yousafzai et al., 2016). To this point, we use structural equation modeling (SEM), a technique that allows the examination of multiple pathways simultaneously to identify both direct and indirect effects of predictors, to test a theoretical model that can also be used to inform interventions aimed at improving child development.

The study site for the current research was rural West Champaran, Bihar. Bihar is one of the poorest states in India. The latest National Family Health Survey (NFHS–4) states that 48% of children under 5 years of age are stunted and 64% are anemic (International Institute of Population Sciences, 2016). Furthermore, over 40% of females over 6 years have never attended school and 42% of households do not have electricity (International Institute of Population Sciences, 2016). Using data from an endline evaluation of a home fortification program with multiple micronutrients in children 12–18 months of age, we used path analysis to examine the cross‐sectional relations among diet, hemoglobin, nutritional status, child engagement, and child development. In a significant extension of the existing literature, we used as outcomes individual child behavioral measures of specific cognitive skills (memory and executive function) in addition to parent‐reported language and personal‐social development. We fitted a single model to the data, allowing for examination of multiple pathways simultaneously, as well as identification of indirect and direct effects of predictors. Moreover, we examined how the magnitude of the pathways between key determinants differed for language and personal‐social development compared to memory and executive function. Key determinants in the analysis included dietary diversity, hemoglobin, LAZ, motor development, and stimulation.

## Hypotheses for Pathways of Interest

**Figure 1:**
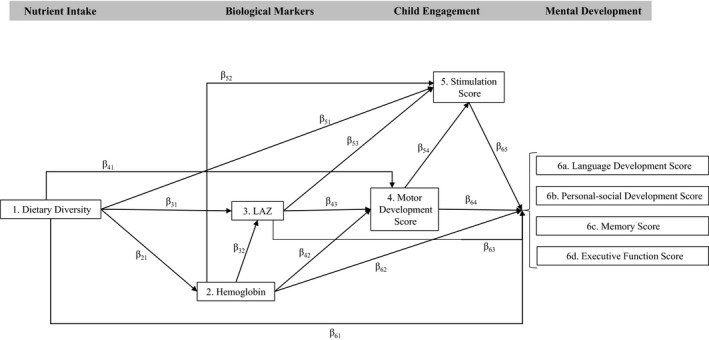
Hypothesized biopsychosocial model. *Note*. All outcomes included in a single model for possible comparison. Length‐for‐age *z*‐score (LAZ).

Many pathways exist between nutrition and early mental development. We sampled broadly across a number of domains of function, including motor, social, and cognitive, and selected measures that, based on prior research, have been found to be predictors of these domains. Our analysis was guided by an hypothesized biopsychosocial model relating nutrient intake to mental development. Here, we outline the hypothesized pathways and their supporting literature (Figure 1). We hypothesized an association between dietary diversity and LAZ (β_31_) based on recent reviews and meta‐analyses that have documented the effects of nutrition on linear growth in children under 5 years of age (Ramakrishnan, Nguyen, & Martorell, 2009; Roberts & Stein, 2017). For instance, a meta‐analysis of randomized controlled trials in preschool age children found a small yet significant effect of multiple micronutrients on linear growth (Ramakrishnan et al., 2009). Diet (β_41_), hemoglobin (β_42_), and growth (β_43_) have been shown to affect a child's level of physical activity (Meeks Gardner, Grantham‐McGregor, Chang, Himes, & Powell, 1995) and the onset of locomotion by improving body size, proportions, mass, and strength (Adolph & Tamis‐LeMonda, 2014; Thelen, 1985). Implications extend to the neurochemistry, neurotransmission, and myelination of neural pathways in the brain (Forssberg, 1985; Pollitt et al., 1994). A meta‐analysis of randomized trials in LMICs, Larson and Yousafzai (2017) found a significant effect of multiple micronutrients on mental development (including cognitive and language abilities; β_61_) in children under 2 years of age. Furthermore, a recent meta‐analysis of preschool‐ and school‐age children from 29 LMICs reported a significant association between height‐for‐age *z*‐scores and motor development (β_43_), as well as cognition (overall domain; β_63_; Sudfeld et al., 2015). We also hypothesized an association among nutrient intake (β_51_), stunting (β_53_), and stimulation. Studies have reported that undernourished children seek more closeness with their mother and engage less often with toys compared to well‐nourished children (Graves, 1978; Lozoff et al., 1998; Sigman & Wachs, 1991; Wachs et al., 1992). Furthermore, a child who is taller, appears older, and is more mobile may demand and receive more stimulation and engagement from others (Brown & Pollitt, 1996). Hemoglobin is highlighted here as a measure of the oxygen carrying capacity of blood to supply the brain and muscles, with potential to improve motor (β_42_) and cognitive abilities (β_62_). Levels of iron in the brain have been shown to influence enzyme systems regulating brain growth, myelination, dopamine receptor synthesis, and energy metabolism in the hippocampus and prefrontal cortex (Beard, 2003; de Ungria et al., 2000; Erikson, Pinero, Connor, & Beard, 1997; Larkin, Jarratt, & Rao, 1986; Lozoff, 2000; Rao, Tkac, Schmidt, & Georgieff, 2011). Lastly, diet (β_61_), LAZ (β_63_), motor development (β_64_), and stimulation (β_65_) are hypothesized to predict mental development and cognitive functioning either directly through brain development (Caldji et al., 1998; Georgieff, 2007; Higley, Suomi, & Linnoila, 1992) or through a child's engagement with other people and their environment, and through attentiveness or neglect from others (Cheatham, Larkina, Bauer, Toth, & Cicchetti, 2010).

Our analysis uses SEM to examine a theoretical model of the associations between diet, hemoglobin, LAZ, motor development, stimulation, and an array of cognitive domains. We use a large cross‐sectional sample of children 12–18 months of age living in rural Bihar, India, a population with high rates of malnutrition. Furthermore, this is a population and age group that, to our knowledge, has not been studied on such a large scale using biological measurements of hemoglobin and LAZ in addition to measurements of social, language, executive function, and memory abilities. As such, the analysis has the potential to extend the literature by examining associations between predictors and various cognitive domains which have been tested through different means (parent report and direct child assessment) and provides the opportunity to examine multiple predictors and their interactions. Further, others have suggested that the use of more general measures of mental development in young children may be masking effects and associations between nutrition and specific cognitive domains (Georgieff, 2007). For instance, Bauer and Dugan (2016) recommend extending to other nutrition research measures of memory function shown to be sensitive to iron deficiency. We begin to address this gap in the literature by testing associations between nutrition‐related predictors and sensitive measures of memory and executive function and comparing them to parent‐reported language and personal‐social development.

## Method

This study was a collaboration between CARE India and Emory University. It was reviewed and approved by the Institutional Review Boards of all partners in the study (3rd Futures Group, Delhi, India, St John's Medical College & Hospital Institutional Ethics Committee, Bangalore, India, and Emory University, Atlanta, USA), and registered with the US National Institute of Health as a clinical trial (http://www.ClinicalTrials.gov; NCT02593136).

### Study Design and Participants

The current analysis used data from an endline evaluation of a cluster randomized trial to examine the effects of a home fortification program with multiple micronutrient powders (MNPs) on anemia, feeding practices, and child development. A full description of the design has been published elsewhere (Larson, 2017). Briefly, frontline health workers delivered either MNPs and nutrition counseling (intervention) or nutrition counseling alone (control) to households with children 6–18 months of age for a period of 12 months. As part of the endline survey, conducted in February–March 2016, a household survey collected data on 2,288 children 12–18 months of age from 70 rural health subcenter communities (HSCs) which had been randomly assigned to intervention or control communities using a simple randomization method with random number generator. In each HSC, 20 of the 31 children were randomly selected to be measured for anthropometry and hemoglobin; 17 of the 20 were also tested for cognitive abilities using direct child assessments, through game‐like tasks (refer to Measurements).

### Measurements

The data collection team included 13 supervisors, 70 household survey data collectors, 32 anthropometry/hemoglobin data collectors, and 18 research assistants for the direct child assessments. Data collectors at endline were staff from CARE India's Bihar Technical Support Program. All had at least completed secondary school and spoke the local language (which was the same across the population sampled). Research assistants conducting direct child assessments were local university graduates in Psychology or Social Science. All data collectors and research assistants were trained in their respective duties over a 2‐week period. The questionnaire and direct child assessment tasks were pilot tested on mothers and children from Bihar to ensure cultural appropriateness. Training on direct child assessments was performed by the first author and a local psychologist. These individuals also monitored administration throughout data collection, to ensure fidelity to the research protocol by all administrators.

Measurements of socio‐demographic characteristics are described in detail elsewhere (Larson, 2017; Larson et al., 2017). A child dietary diversity score was created according to WHO guidelines, using number of food groups out of seven consumed in the past day (WHO, 2008). Length was measured with the Seca 417; LAZ were calculated using the WHO 2006 child growth standards (de Onis, Onyango, Van den Broeck, Chumlea, & Martorell, 2004). Hemoglobin was measured with the HemoCue Hb 201+ Analyzar (HemoCue, Angelholm, Sweden) using capillary blood from a finger prick. Family care indicators (FCI), a nine‐item parent‐report measure, previously validated in South Asia (Hamadani et al., 2010), was used to assess psychosocial stimulation.

#### Developmental Milestones

Child development was assessed using the Developmental Milestones Checklist, 2nd ed. (DMC–II; (Prado et al., 2014), a 75‐item parent report of gross and fine motor (32 items; e.g., “Child walks alone five steps”), language (15 items; e.g., “Child uses gestures to communicate”), and personal‐social (28 items on reaction to others, recognition of others, play, dressing, eating and drinking, and toilet training) development. Motor development includes the sum of scores from the gross and fine motor subscales. Items were scored as 1 if the child had performed this activity and 0 if the child had not yet performed it. This measure has been validated in India, Burkina Faso, Kenya, and Ghana (Abubakar, Holding, Van de Vijver, Bomu, & Van Baar, 2010; Ahun, 2015; Larson et al., 2017; Prado et al., 2014).

#### Memory Test

Episodic memory was measured using elicited and deferred imitation (Bauer, 2005, 2010). Three‐dimensional props are used to demonstrate a specific sequence of actions that the child is then invited to imitate either immediately or after a delay. Each child was tested for immediate and delayed recall of sequences two and three steps in length. Specifically, each child was tested on 1 two‐step sequence and 1 three‐step sequence immediately after modeling (immediate recall) and 1 two‐step and 1 three‐step sequence after a delay of 10 min (different sequences were used to test immediate and delayed recall). Research assistants worked in pairs; one modeled the sequences and interacted with the child and the other took notes and scored the children's performance. For a complete description of the elicited and deferred imitation task, refer to Larson (2017). To score the children's behavior, for each sequence, we calculated a total number of individual target actions produced (hence referred to as target actions; maximum = 2 for two‐step sequences, maximum = 3 for three‐step sequences) and the total number of pairs of actions produced in the target order (hence referred to as ordered recall; maximum = 1 for two‐sequence tasks, maximum = 2 for three‐sequence tasks).

#### Executive Function

Executive function was measured using the A‐not‐B task (Diamond, 1985). The child is shown a desirable object hidden under a cloth (location A), and after a brief delay, the child is allowed to search and find the object. After several successes finding the object at a particular location, the object is then hidden under a cloth at an alternate location (B). For a complete description of the A‐not‐B task, refer to Larson (2017). Children were scored as making an error if they reached to the empty cloth, if they did not reach at all over the course of 30 s, or if they reached simultaneously to both cloths. The first reversal trial had no delay. No reversal trial was administered until the child reached correctly on the two trials prior to the reversal. Each child was given four trial attempts to retrieve the object successfully under a given delay. If the child was successful in retrieving the object on two consecutive trials, the side of hiding was changed and the delay incremented by 3 s. This was continued until the child failed to retrieve the object on two consecutive trials or the maximum of 12 s delay was successfully passed (delays: 0, 3, 6, 9, 12 s). To score the child's behavior, we noted perseverative error (not finding the toy under cloth B after finding toy under cloth A) and maximum tolerated delay in seconds (none, 3, 6, 9, or 12).

### Statistical Analyses

Children 12–18 months of age were randomly selected from the full survey to receive all three developmental assessments, the DMC–II, the Elicited Imitation task, and the A‐not‐B test (*N* = 1,079 or 47% of the full sample). Children randomly selected for additional cognitive testing did not differ from those included in the full sample on any of the demographic or clinical characteristics. Univariate analyses were used to examine the distributions of statistical predictors and outcome variables and test for normality. Means, standard deviations, frequencies, and counts were determined for household, maternal, and child characteristics of relevance to the outcome, and the outcomes themselves. We examined the correlation matrix to identify significant correlations between exogenous and endogenous variables. Bivariate analyses informed the relations between outcomes and each statistical predictor in the hypothesized model. The model was derived from Pollitt's (2000) original framework.

Univariate and bivariate analyses were conducted using SAS version 9.4 (SAS Institute, Cary, NC). The structural equation model was developed in MPlus version 7 (Muthén & Muthén, Los Angeles, CA) using weighted least square means with missing values estimation. The hypothesized path model examined the direct and indirect associations among child dietary diversity, hemoglobin concentration, LAZ, motor development score (sum of gross and fine motor development subscales of the DMC–II), stimulation score, and language and personal‐social development scores, memory, and executive function outcomes. A single model was fitted to the data.

Model fit was evaluated with common standards: a comparative fit index (CFI; Bentler, 1990) and a Tucker–Lewis index (TLI; Tucker & Lewis, 1973) > 0.90 for acceptable fit and > 0.95 for good fit; and a root mean square error of approximation (RMSEA; Steiger, 1990) < .08 for acceptable fit and < .05 for good fit. If model fit statistics were not acceptable, modification indices were examined to decide whether additional pathways should be examined to improve model fit, and the model was re‐specified.

The following variables were examined as potential covariates or confounders in our model: age of child in months, child sex, intervention group, wealth quintile of the household, parity (number of times mother has given live birth), illness, caste, young mother (defined as < 18 years of age at first birth), and maternal education (dichotomized as no vs. any school attendance). All analyses were adjusted for clustering at the HSC level. The outcomes of interest using the DMC–II test were language and personal‐social development scores. Outcomes from the Elicited Imitation tasks included (a) target actions completed in all sequences (continuous), and (b) ordered recall completed in all sequences (continuous). All sequence scores were summed because there was no significant difference in the outcomes for sequences performed with or without the 10‐min delay. Outcomes of interest on the A‐not‐B task were (a) ability to find the object under cloth B (overcoming perseverative error), and (b) ability to tolerate 3, 6, 9, and 12 s, or not tolerating any delay. We grouped all children who tolerated any delay because a small proportion (22%) of children had a maximum tolerated delay between 0 and 12 s (i.e., maximum tolerated delay of 3, 6, or 9 s). The majority of children who were able to tolerate any delay were able to tolerate a 12 s delay.

Estimates for model pathways were compared to one another and significant differences were established using Wald's chi‐square test.

## Results

Continuous predictors and outcome variables were normally distributed. Twelve children were missing any value for the predictors of interest; all available data were used to estimate the model. No significant differences between children who had missing compared to no missing data were found for language, personal‐social development, memory, executive function, maternal education, wealth quintile, age of child, sex, religion, or caste. The interrater reliability measurements for the DMC–II gave an average kappa coefficient of .96 and no single rater's kappa coefficient was below .70. Internal reliability estimates for the DMC–II were acceptable, with Cronbach's alpha of .92 for the total score, .88 for motor, .65 for language, .80 for personal‐social, and .71 for cognitive subscales. Cronbach's alpha for the FCI score was .60, which is typical given that questions target different ways of providing stimulation and different domains of development (Bornstein, Putnick, Lansford, Deater‐Deckard, & Bradley, 2015).

The mean age of children was 14.6 months, and the sample included fewer girls than boys (Table 1). Almost half of children had experienced either fever, cough, or diarrhea in the 2 weeks prior to the survey; dietary diversity scores were low, with a mean below the acceptable minimum of four categories (Table 1; WHO, 2008). Bivariate regression analyses indicated that dietary diversity, LAZ, stimulation score, and motor development score were significantly associated with language and personal social‐development (Table 2). Hemoglobin was associated with memory and executive function scores, but dietary diversity and stimulation were not (Table 2). Although dietary diversity and stimulation were not associated with these specific cognitive functions, they were included in the modeling effort (see following), due to their theoretical significance. Child age in months, household wealth quintile, maternal education, child sex, religion, and caste were identified as potential confounders because they were significantly associated with at least one outcome and predictive variable. The magnitude of the associations did not differ significantly before and after adjustment for confounding variables. The correlation matrix shows that language and personal‐social development scores were highly intercorrelated, as was motor development with language, personal‐social development, memory, and LAZ (Table 3).

**Table 1:** Demographic and Clinical Characteristics of Children 12–18 Months of Age With Measures of Child Development (*N* = 1,079)

	Children 12–18 months of age
Age of child in months	14.6 ± 1.7
Intervention group assignment	50.2 (542)
Girls	48.1 (519)
Maternal education	
Any schooling	43.1 (465)
Parity	2.7 ± 1.7
Young mother	60.6 (653)
Religion	
Hindu	79.3 (844)
Muslim	21.7 (234)
Caste	
Scheduled caste	20.9 (226)
Scheduled tribe	9.2 (99)
Other backwards caste	10.6 (114)
Household wealth index (quintile)	3.0 ± 1.4
Recent illness	
Any	48.3 (521)
Fever	32.9 (355)
Cough	35.8 (386)
Diarrhea	11.6 (125)
Child nutrition and engagement	
Dietary diversity score (out of 7)	3.4 ± 1.2
Family care indicators score (out of 9)	5.6 ± 1.7
Hemoglobin concentration (g/dl)	10.4 ± 1.3
Length‐for‐age *z*‐score	−1.7 ± 1.1
Child development	
Motor development score (out of 32)	23.4 ± 4.2
Language development score (out of 15)	6.7 ± 2.1
Personal‐social development score (out of 28)	18.8 ± 2.8
Memory score (ordered recall; out of 6)	1.8 ± 1.2
Memory score (target actions; out of 10)	5.0 ± 1.9
Overcame perseverative error	69.5 (742)
Any tolerated delay	59.6 (636)

Note

Values are % (*N*) or *M* ± *SD*. All estimates account for cluster‐randomization by health subcenter.

**Table 2:** Bivariate Analyses Examining Predictors of Language, Personal‐Social, Memory, and Executive Function

	Unadjusted	*p*‐Value	Adjusted	*p*‐Value
Language development score				
Dietary diversity	.10 (−.03, .23)	.138	.05 (−.05, .15)	.315
Hemoglobin	.17 (.06, .28)	.003	.12 (.01, .22)	.035
Length‐for‐age *z*‐score	.34 (.20, .48)	< .001	.37 (.24, .50)	< .001
Motor development score	.29 (.26, .31)	< .001	.23 (.20, .27)	< .001
Stimulation score	.31 (.22, .39)	< .001	.24 (.15, .32)	< .001
Personal‐social development score				
Dietary diversity	.16 (.01, .32)	.040	.11 (−.01, .23)	.082
Hemoglobin	.10 (−.03, .24)	.125	.04 (−.09, .16)	.559
Length‐for‐age *z*‐score	.41 (.23, .59)	< .001	.44 (.27, .60)	< .001
Motor development score	.38 (.35, .41)	< .001	.33 (.29, .37)	< .001
Stimulation score	.24 (.14, .34)	< .001	.14 (.03, .24)	.012
Memory score (ordered recall)				
Dietary diversity	.01 (−.07, .09)	.859	.00 (−.08, .07)	.921
Hemoglobin	.10 (.04, .15)	.001	.07 (.03, .12)	.004
Length‐for‐age *z*‐score	.09 (.01, .16)	.026	.10 (.02, .17)	.011
Motor development score	.07 (.06, .09)	< .001	.04 (.03, .06)	< .001
Stimulation score	.01 (−.04, .06)	.742	−.02 (−.07, .03)	.343
Memory score (target actions)				
Dietary diversity	.02 (−.10, .15)	.701	.01 (−.12, .13)	.928
Hemoglobin	.11 (.01, .20)	.036	.08 (−.01, .18)	.076
Length‐for‐age *z*‐score	.13 (.01, .26)	.034	.14 (.02, .26)	.019
Motor development score	.12 (.09, .15)	<.001	.09 (.06, .12)	<.001
Stimulation score	.06 (−.02, .15)	.136	.02 (−.07, .11)	.628
Executive function score (overcome perseverative error)				
Dietary diversity	.01 (−.02, .04)	.398	.01 (−.02, .04)	.411
Hemoglobin	.02 (.00, .05)	.021	.02 (.00, .04)	.028
Length‐for‐age *z*‐score	.01 (−.01, .04)	.273	.01 (−.01, .04)	.307
Motor development score	.01 (.01, .02)	.001	.01 (.00, .01)	.048
Stimulation score	.02 (.00, .03)	.062	.01 (−.01, .03)	.165
Executive function score (any tolerated delay)				
Dietary diversity	.01 (−.02, .04)	.514	.01 (−.02, .04)	.482
Hemoglobin	.03 (.00, .05)	.040	.02 (.00, .04)	.092
Length‐for‐age *z*‐score	.03 (.00, .05)	.048	.02 (−.01, .04)	.126
Motor development score	.01 (.00, .02)	.001	.01 (.00, .01)	.084
Stimulation score	.01 (−.01, .03)	.180	.00 (−.02, .03)	.624

Note

Values are β coefficients (95% CI). All adjusted for clustering at the health subcenter level. Adjusted analyses accounting for age of child in months, child sex, intervention group, wealth quintile of the household, religion, caste, and maternal education.

**Table 3:** Correlation Matrix Between Predictors and Mental Development

	Personal‐social development	Executive function (overcome perseverative error)	Executive function (tolerate any delay)	Memory (target actions)	Memory (ordered recall)	Dietary diversity score	Hemoglobin concentration	Length‐for‐age *z*‐score	Stimulation score	Motor development
Language development	.60 (< .0001)	.06 (.0518)	.08 (.0142)	.20 (< .0001)	.22 (< .0001)	.05 (.0825)	.11 (.0006)	.18 (< .0001)	.24 (< .0001)	.56 (< .0001)
Personal‐social development		.05 (.1228)	.07 (.0218)	.20 (< .0001)	.19 (< .0001)	.07 (.0259)	.05 (.0959)	.17 (< .0001)	.15 (< .0001)	.58 (< .0001)
Executive function (overcome perseverative error)			.79 (< .0001)	.30 (< .0001)	.24 (< .0001)	.03 (.3561)	.07 (.0208)	.03 (.2744)	.06 (.0581)	.11 (.0005)
Executive function (tolerate any delay)				.30 (< .0001)	.24 (< .0001)	.02 (.4262)	.07 (.0208)	.06 (.0594)	.04 (.1458)	.10 (.0009)
Memory (target actions)					.84 (< .0001)	.02 (.6342)	.08 (.0202)	.08 (.0143)	.06 (.0735)	.28 (< .0001)
Memory (ordered recall)						.01 (.8281)	.11 (.0008)	.08 (.021)	.01 (.7205)	.26 (< .0001)
Dietary diversity score							−.01 (.7389)	−.01 (.687)	.05 (.0972)	.04 (.2466)
Hemoglobin concentration								.07 (.0287)	.06 (.0364)	.10 (.0007)
Length‐for‐age *z*‐score									.14 (< .0001)	.26 (< .0001)
Stimulation score										.16 (< .0001)

Note

Values are Pearson or Spearman rank correlation coefficients (*p*‐value).

**Figure 2:**
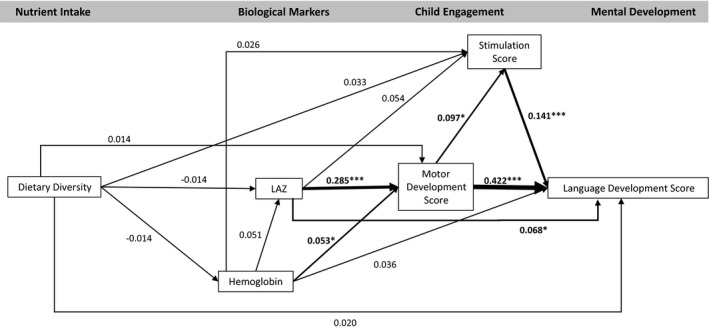
Standardized coefficients in path model between diet and language development of children 12–18 months of age (*N* = 1,079). *Note*. Analysis adjusted for age of child, intervention group, wealth quintile, maternal education, child sex, religion, and caste, and accounted for clustering at the health subcenter level. Analysis used weighted least square means with missing values. Length‐for‐age *z*‐score (LAZ). *p < .05. ***p < .001.

**Figure 3:**
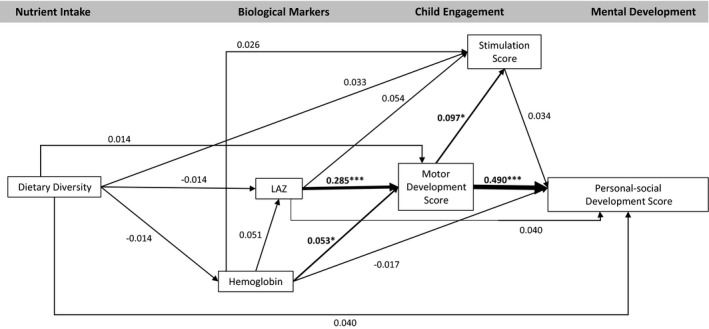
Standardized coefficients in path model between diet and personal‐social development of children 12–18 months of age (*N* = 1,079). *Note*. Analysis adjusted for age of child, intervention group, wealth quintile, maternal education, child sex, religion, and caste, and accounted for clustering at the health subcenter level. Analysis used weighted least square means with missing values. Length‐for‐age *z*‐score (LAZ). *p < .05. ***p < .001.

**Figure 4:**
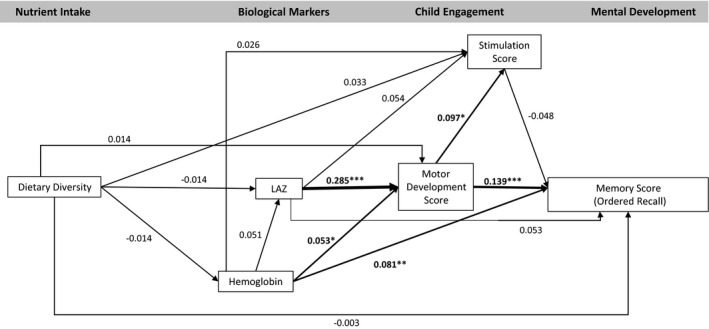
Standardized coefficients in path model between diet and memory score (ordered recall) of children 12–18 months of age (*N* = 1,079). *Note*. Analysis adjusted for age of child, intervention group, wealth quintile, maternal education, child sex, religion, and caste, and accounted for clustering at the health subcenter level. Analysis used weighted least square means with missing values. Length‐for‐age *z*‐score (LAZ). *p < .05. **p < .01. ***p < .001.

**Figure 5:**
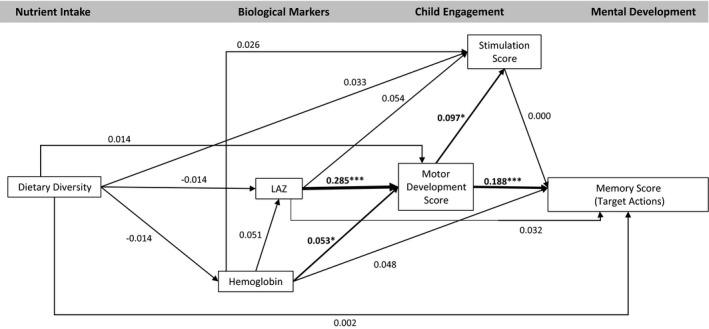
Standardized coefficients in path model between diet and memory score (target actions) of children 12–18 months of age (*N* = 1,079). *Note*. Analysis adjusted for age of child, intervention group, wealth quintile, maternal education, child sex, religion, and caste, and accounted for clustering at the health subcenter level. Analysis used weighted least square means with missing values. Length‐for‐age *z*‐score (LAZ). *p < .05. ***p < .001.

**Figure 6:**
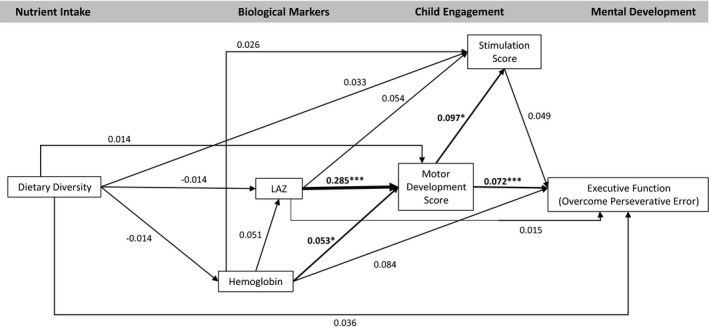
Standardized coefficients in path model between diet and executive function (overcome perseverative error) of children 12–18 months of age (*N* = 1,079). *Note*. Analysis adjusted for age of child, intervention group, wealth quintile, maternal education, child sex, religion, and caste, and accounted for clustering at the health subcenter level. Analysis used weighted least square means with missing values. Length‐for‐age *z*‐score (LAZ). *p < .05. ***p < .001.

**Figure 7:**
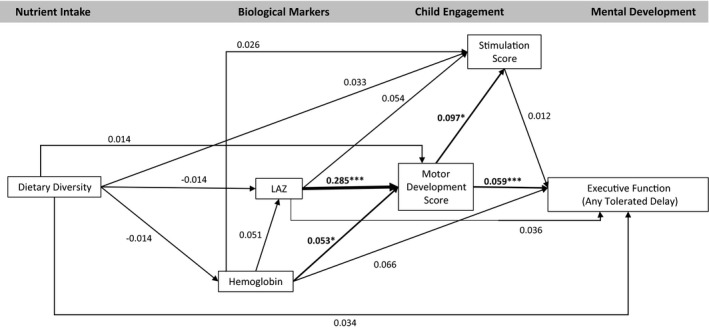
Standardized coefficients in path model between diet and executive function (any tolerated delay) of children 12–18 months of age (*N* = 1,079). *Note*. Analysis adjusted for age of child, intervention group, wealth quintile, maternal education, child sex, religion, and caste, and accounted for clustering at the health subcenter level. Analysis used weighted least square means with missing values. Length‐for‐age *z*‐score (LAZ). *p < .05. ***p < .001.

The model fit statistics confirmed the data fit the model (CFI = 1.000, TLI = 1.076, RMSEA < .001). The model indicated no significant association between dietary diversity and any endogenous variables (hemoglobin, LAZ, motor development, stimulation, or any development outcomes; Figures 2, 3, 4, 5, 6, 7). LAZ was moderately and significantly associated with motor development, standardized β (β) = .285, *p* < .001; hemoglobin was modestly associated with motor development (β = .053, *p* = .046); and motor development was modestly associated with stimulation (β = .097, *p* = .029; Figures 2, 3, 4, 5, 6, 7). Motor development was strongly and significantly associated with language development (β = .422, *p* < .001; Figure 2) and with personal‐social development (β = .490, *p* < .001; Figure 3). Motor development was moderately associated with memory scores and executive function scores (Figures 4, 5, 6, 7). The associations between motor and language and between motor and personal‐social scores were significantly larger than the associations between motor and memory and between motor and executive function scores (Wald test *p* < .001 for all). Stimulation was significantly associated with language development (Figure 2); this association was significantly smaller than the association between motor and language development, but larger than the association between LAZ and language development (Wald test *p* < .001 for all). Of all the child development outcomes, hemoglobin was significantly associated with only the memory outcome of ordered recall.

Significant indirect effects were observed for hemoglobin on motor, language, personal social, and memory (target actions) score (Tables S1 and S2). Significant indirect effects were observed for LAZ on stimulation, language, personal‐social, and memory scores (Tables S1 and S2). *R* ^2^ values are shown (Table S3).

## Discussion

Our findings begin to fill a gap in evidence on nutritional and psychosocial predictors of parent‐reported language and personal‐social development, compared to sensitive measures of memory and executive function in an understudying population and age group. The current path analysis examined direct and indirect associations between nutrient intake, biological markers, and child engagement, and outcomes of language, personal‐social development, memory, and executive function in children 12–18 months of age living in rural Bihar. The framework used was adapted from Pollitt's (2000) work to include hemoglobin and examine differences in the pathways for a range of cognitive outcomes, controlling for age and other confounding variables. The significant associations between LAZ, hemoglobin, motor, stimulation, and cognitive outcomes add to previous models by examining associations with parental report measures of development, as well as behavioral measures of specific cognitive functions.

The strongest and most consistent pattern observed was the direct and indirect relation between LAZ, motor development and mental development, including personal‐social development, memory, and executive function, but especially language development. Similar significant associations between LAZ and motor development, and LAZ and language development, have been reported in a study of children 5–19 months of age living in Zanzibar (Olney et al., 2009). Direct associations between linear growth and motor and language development have also been reported in a study of children from Ghana, Malawi, and Burkina Faso (Prado et al., 2017). Strength, muscle mass, and endurance are important in children 6–24 months of age who are learning to crawl, walk, and run (Adolph, Vereijken, & Denny, 1998). Additionally, a child who appears older may be engaged by their parents and adults more often and with more stimulating language than a child who looks younger. Importantly, the benefits of motor development on mental development accrue only if they lead to richer experiences, more objects to play with, and stimulating situations and interactions with others (both gross and fine motor abilities; Adolph & Tamis‐LeMonda, 2014). This may depend on the type of nutrition provided. For instance, Aburto et al. found that child supplementation for 4 months with macro‐ and micronutrients enhanced the level of exploration (i.e., touching and manipulation of objects, fine motor abilities), whereas supplementation with only multiple micronutrients increased the level of activity performed (i.e., gross motor movements; Aburto, Ramirez‐Zea, Neufeld, & Flores‐Ayala, 2010). The differences Aburto et al. captured could be due, in part, to their use of direct observation measurements of children's motor development, a concept which should be considered in future mediation analyses.

An association often noted (Bauer, 2010; Hamadani, Huda, Khatun, & Grantham‐McGregor, 2006) and reinforced in our study is the relation between stimulation and mental development; however, in our case, it is confined to language development. The type of stimulation assessed (i.e., reading and play materials available, interaction, singing, and story‐telling with others, and time spent naming, counting, etc.) may be less relevant to memory and executive function. Motor development is presented in our study as a predictor of level of child stimulation with the understanding that a child's mobility and fine movements may improve the amount and quality of stimulation provided. Yet, the reverse association may also be applicable, in that children who are given more stimulating material will have more opportunity to develop their motor skills (particularly object manipulation).

A weak, but significant, direct or indirect (through motor development) association with hemoglobin was observed for language, personal‐social development, and memory. Others have also reported significant associations between hemoglobin and motor development (Olney et al., 2009, 2013; Prado et al., 2017). Research in Chilean children showed that greater severity of anemia and duration of anemia of more than 3 months was associated with decreased psychomotor development scores (Walter, 1989). Anemia in children can cause lethargy, reduced attention, and reduced responsiveness to peers resulting in fewer interactions and slower exploration of their environment (Black, Quigg, Hurley, & Pepper, 2011; Walter, 1989). Similar to our findings, no significant association was seen between hemoglobin and language development in Zanzibari children (Olney et al., 2009, 2013). On the other hand, a combined analysis of children from three African countries reported significant associations between hemoglobin at 6 months of age, as well as hemoglobin change from 6 to 18 months of age, and language development at 18 months of age (Prado et al., 2017). The measurement of language development in two of the three countries was performed using a parent‐reported 100‐word vocabulary checklist (Prado et al., 2017), different from the DMC–II used in this study which also includes measures of receptive language.

There was no evidence in our study for an association between diet and LAZ or hemoglobin, as has been shown previously (Wright, Bentley, Mendez, & Adair, 2015), which could be a function of the dietary diversity being a relatively rough measure of nutrient intake. Dietary intake can vary between seasons in rural Bihar, and may not reflect the longer term influences on linear growth. The lack of association could also be due to the role of infection (inflammatory biomarkers not measured here) and its effect on growth and nutritional biomarkers (Namaste, Aaron, Varadhan, Peerson, & Suchdev, 2017).

Our study demonstrates two important findings with respect to memory: hemoglobin concentration is an important statistical predictor of memory (more so than for general cognitive abilities and executive function), and ordered recall in the elicited and deferred imitation task may be a more sensitive measure of memory than the number of target actions completed. Others have found similar relations between hemoglobin and memory (DeBoer, Wewerka, Bauer, Georgieff, & Nelson, 2005; Eilander et al., 2010; Riggins, Miller, Bauer, Georgieff, & Nelson, 2009; Virues‐Ortega et al., 2011). For instance, Eilander et al. (2010) reported that hemoglobin was positively associated with memory in Indian school‐age children. The associations observed could be due to the effects of cerebral blood flow (Hill et al., 2006) and improved function of areas of the brain important for memory (the hippocampus, cerebral cortex, and striatum; McDonough, Mandler, McKee, & Squire, 1995; Nelson & Silverstein, 1994). A study of infants of diabetic mothers, exposed to hypoxic conditions and iron deficiency prenatally, showed decreased recall abilities at 1 year of age compared to children of nondiabetic mothers using the elicited and deferred imitation task (Riggins, Bauer, Georgieff, & Nelson, 2010). Similar to our findings, differences were observed in ordered recall but not target actions. As in Riggins et al. (Riggins et al., 2010), we argue that the specificity of the relation reflects the greater memory demand imposed when reproducing an ordered sequence of actions in the absence of perceptual support, versus performing individual actions that are cued by the objects on which they are performed. In the present research, infants’ performance at immediate and delayed recall did not differ. In contrast, in Riggins et al. (Riggins et al., 2010), for infants of diabetic mothers, ordered recall was significantly lower at delayed recall than at immediate recall, a finding attributed to likely hippocampal impairment in the sample (Reed & Squire, 1998). Consistent with this suggestion, delayed recall at 1 year was marginally correlated with newborn ferritin concentrations (Riggins et al., 2010). Iron deficiency is an important risk factor for anemia in our population (Kumar, Taneja, Yajnik, Bhandari, & Strand, 2014) and may contribute to the relation observed with memory.

The only significant association with executive function was motor development. Other nutrition trials, published after the completion of our study, have shown a similar lack of relation with nutrition as measured using the A‐not‐B task in this same age group (Matias et al., 2017; Prado et al., 2016). The A‐not‐B task has been shown to be sensitive to poverty (Lipina, Martelli, Vuelta, & Colombo, 2005) and other health exposures, such as phenylketonuria (Diamond, Prevor, Callender, & Druin, 1997) and maternal drug abuse during pregnancy (Noland, Singer, Mehta, & Super, 2003). However, aspects of executive function measured by the A‐not‐B task may not be the ones that are impacted by nutrition at this particular point in development. Benefits of nutrition on executive function as measured here may become more apparent later in life when areas of the brain (especially the prefrontal cortex) develop more fully (Diamond & Ling, 2016).

Limitations of this analysis include the cross‐sectional nature of the associations. The relations observed cannot be taken as causal, and as mentioned, the direction of some associations presented could be reversed. Dietary diversity is a rough measure of food groups consumed over the previous day, and does not approximate nutrient or food intake. Furthermore, the inclusion of other nutritional and infection biomarker measurements, such as ferritin, C‐reactive protein, and alpha1‐acid‐glycoprotein, or information on breastfeeding initiation and duration may have strengthened our model. Lastly, measurement of motor, language, and personal‐social development were parent‐reported and could be biased by the respondent.

On the other hand, memory and executive function were measured through interactions with the children themselves which would minimize caretaker bias. Other strengths of the study include a large sample size, rigorous sampling of an at‐risk population, and the measurement of a range of child development outcomes. To our knowledge, this is the first study to use the elicited and deferred imitation task to examine memory in young children living in South Asia.

In conclusion, our findings inform the development of children in this population, and could contribute to the design of interventions to improve child development in this context. A path analysis examined the relations between nutrient intake, biological markers and child engagement and parental report measures of general cognitive function, as well as behavioral measures of memory and executive function. The strong direct and indirect relations observed between LAZ, motor development and cognitive abilities highlights their importance in this population. Stimulation has a significant association with language development, as does hemoglobin with memory.

## Supporting information


**Table S1.** Standardized Direct, Indirect, and Total Effects for Path Model
**Table S2.** Unstandardized Direct, Indirect, and Total Effects for Path Model
**Table S3.** *R* ^2^ Values for Path Model
