# Supplementary material for: A Path Analysis of Nutrition, Stimulation, and Child Development Among Young Children in Bihar, India
Source: Child Dev. 2018 Mar 12;89(5):1871–86. doi: 10.1111/cdev.13057 (PMC6174960; doi:10.1111/cdev.13057)
Supplement: Supplementary file 1 — Table S1. Standardized Direct, Indirect, and Total Effects for Path Model Table S2. Unstandardized Direct, Indirect, and Total Effects for Path Model Table S3. R 2 Values for Path Model [file CDEV-89-1871-s001.docx]

Supplemental Table 1: Standardized direct, indirect, and total effects for path model^1^

|  |  |  | **Standardized Coefficient (95% CI)** | | |
| --- | --- | --- | --- | --- | --- |
| **Dependent variables** | **Predictors** |  | **Direct Effect** | **Indirect effect** | **Total effect** |
| Hemoglobin concentration | Dietary diversity | | -0.014 (0.052, 0.024) |  |  |
| Length-for-age z-score | Dietary diversity | | -0.014 (-0.071, 0.042) | -0.001 (-0.003, 0.001) | -0.015 (-0.071, 0.041) |
|  | Hemoglobin concentration | | 0.051 (-0.002, 0.105) |  |  |
| Motor development score | Dietary diversity | | 0.014 (-0.022, 0.050) | -0.005(-0.021, 0.011) | 0.009 (-0.033, 0.050) |
|  | Hemoglobin concentration | | 0.053 (0.010, 0.097)* | 0.015 (0.000, 0.029) | 0.068 (0.018, 0.118)* |
|  | Length-for-age z-score | | 0.285 (0.238, 0.332)*** |  |  |
| Stimulation score | Dietary diversity | | 0.048 (-0.024, 0.121) | 0.000 (-0.007, 0.006) | 0.033 (-0.019, 0.085) |
|  | Hemoglobin concentration | | 0.026 (-0.028, 0.080) | 0.009 (0.003, 0.016)* | 0.036 (-0.019, 0.090)) |
|  | Length-for-age z-score | | 0.054 (-0.021, 0.130) | 0.028 (0.007, 0.048)* | 0.082 (0.007, 0.156) |
|  | Motor development score | | 0.097 (0.025, 0.169)* |  |  |
| Language development score | Dietary diversity | | 0.020 (-0.019, 0.060) | 0.007 (-0.016, 0.030) | 0.027 (-0.020, 0.074) |
|  | Hemoglobin concentration | | 0.036 (-0.009, 0.081) | 0.037 (0.012, 0.063)* | 0.074 (0.016, 0.131)* |
|  | Length-for-age z-score | | 0.068 (0.021, 0.115)* | 0.132 (0.098, 0.166)*** | 0.200 (0.134, 0.265)*** |
|  | Motor development score | | 0.422 (0.368, 0.475)*** | 0.014 (0.002, 0.025) | 0.435 (0.379, 0.491)*** |
| Personal-social development score | Dietary diversity | | 0.040 (0.007, 0.074) | 0.005 (-0.017, 0.027) | 0.045 (0.007, 0.084) |
|  | Hemoglobin concentration | | -0.017 (-0.061, 0.026) | 0.037 (0.011, 0.062)* | 0.019 (-0.032, 0.071) |
|  | Length-for-age z-score | | 0.040 (-0.012, 0.092) | 0.143 (0.115, 0.170)*** | 0.183 (0.123, 0.242)*** |
|  | Motor development score | | 0.490 (0.449, 0.531)*** | 0.003 (-0.002, 0.009) | 0.493 (0.452, 0.534)*** |
| Memory score (ordered recall) | Dietary diversity | | -0.003 (-0.064, 0.058) | -0.002 (-0.010, 0.006) | -0.006 (-0.068, 0.057) |
|  | Hemoglobin concentration | | 0.081 (0.037, 0.125)** | 0.010 (0.001, 0.020) | 0.091 (0.046, 0.137)** |
|  | Length-for-age z-score | | 0.053 (-0.005, 0.112) | 0.036 (0.017, 0.055)** | 0.089 (0.030, 0.148)* |
|  | Motor development score | | 0.139 (0.081, 0.197)*** | -0.005 (-0.012, 0.002) | 0.134 (0.075, 0.193)*** |
| Memory score (target actions) | Dietary diversity | | 0.002 (-0.060, 0.064) | 0.000 (-0.009, 0.010) | 0.002 (-0.061, 0.066) |
|  | Hemoglobin concentration | | 0.048 (-0.007, 0.104) | 0.014 (0.003, 0.026)* | 0.063 (0.006, 0.120) |
|  | Length-for-age z-score | | 0.032 (-0.026, 0.090) | 0.054 (0.033, 0.074)*** | 0.086 (0.026, 0.146)* |
|  | Motor development score | | 0.188 (0.131, 0.245)*** | 0 | 0.188 (0.130, 0.246)*** |
| Executive function (Overcome perseverative error) | Dietary diversity | | 0.036 (-0.033, 0.104) | 0.001 (-0.005, 0.007) | 0.036 (-0.034, 0.106) |
|  | Hemoglobin concentration | | 0.084 (0.013, 0.156) | 0.007 (0.000, 0.015) | 0.092 (0.023, 0.161)* |
|  | Length-for-age z-score | | 0.015 (-0.064, 0.095) | 0.025 (-0.001, 0.050) | 0.040 (-0.047, 0.127) |
|  | Motor development score | | 0.015 (-0.064, 0.095) | 0.005 (-0.004, 0.013) | 0.077 (-0.003, 0.157) |
| Executive function (Any tolerated delay) | Dietary diversity | | 0.034 (-0.040, 0.107) | -0.001 (-0.006, 0.004) | 0.033 (-0.042, 0.108) |
|  | Hemoglobin concentration | | 0.066 (-0.005, 0.136) | 0.006 (0.000, 0.013) | 0.072 (0.002, 0.142) |
|  | Length-for-age z-score | | 0.036 (-0.036, 0.109) | 0.018 (-0.004, 0.040) | 0.054 (-0.018, 0.127) |
|  | Motor development score | | 0.059 (-0.015, 0.133) | 0.001 (-0.006, 0.008) | 0.060 (-0.013, 0.134) |
| ^1^Values are β coefficients (95% CI). All adjusted for clustering at the health sub-center level. Adjusted analyses accounting for age of child in months, child sex, intervention group, wealth quintile of the household, religion, caste, and maternal education. Estimates of zero are not shown. * P<0.05; ** P<0.01; *** P<0.001. | | | | | |

Supplemental Table 2: Unstandardized direct, indirect, and total effects for path model^1^

|  |  |  | | **Unstandardized Coefficient (95% CI)** | | |
| --- | --- | --- | --- | --- | --- | --- |
| **Dependent variables** | **Predictors** |  | | **Direct Effect** | **Indirect effect** | **Total effect** |
| Hemoglobin concentration | Dietary diversity | | -0.016 (0.061, 0.028) | |  |  |
| Length-for-age z-score | Dietary diversity | | -0.014 (-0.069, 0.041) | | -0.001 (-0.003, 0.001) | -0.014 (-0.069, 0.040) |
|  | Hemoglobin concentration | | 0.043 (-0.002, 0.088) | |  |  |
| Motor development score | Dietary diversity | | 0.050 (-0.081, 0.181) | | -0.018 (0.078, 0.042) | 0.032 (-0.120, 0183) |
|  | Hemoglobin concentration | | 0.167 (0.029, 0.305)* | | 0.046 (0.001, 0.091) | 0.213 (0.055, 0.371)* |
|  | Length-for-age z-score | | 1.070 (0.874, 1.266)*** | |  |  |
| Stimulation score | Dietary diversity | | 0.048 (-0.024, 0.121) | | 0.000 (-0.010, 0.009) | 0.048 (-0.027, 0.123) |
|  | Hemoglobin concentration | | 0.033 (-0.034, 0.099) | | 0.012 (0.004, 0.019)* | 0.044 (-0.023, 0.112)) |
|  | Length-for-age z-score | | 0.081 (-0.033, 0.195) | | 0.041 (0.010, 0.072)* | 0.122 (0.009, 0.235) |
|  | Motor development score | | 0.038 (0.009, 0.067)* | |  |  |
| Language development score | Dietary diversity | | 0.038 (-0.036, 0.112) | | 0.013 (-0.030, 0.055) | 0.051 (-0.038, 0.139) |
|  | Hemoglobin concentration | | 0.058 (-0.015, 0.131) | | 0.059 (0.018, 0.101)* | 0.118 (0.024, 0.211)* |
|  | Length-for-age z-score | | 0.130 (0.041, 0.219)* | | 0.252 (0.186, 0.317)*** | 0.381 (0.256, 0.507)*** |
|  | Motor development score | | 0.215 (0.187, 0.243)*** | | 0.007 (0.001, 0.013) | 0.222 (0.192, 0.251)*** |
| Personal-social development score | Dietary diversity | | 0.097 (0.015, 0.179) | | 0.012 (-0.041, 0.065) | 0.109 (0.016, 0.202) |
|  | Hemoglobin concentration | | -0.036 (-0.125, 0.054) | | 0.075 (0.021, 0.129)* | 0.040 (-0.067, 0.146) |
|  | Length-for-age z-score | | 0.099 (-0.028, 0.226) | | 0.350 (0.280, 0.420)*** | 0.449 (0.303, 0.596)*** |
|  | Motor development score | | 0.321 (0.293, 0.349)*** | | 0.002 (-0.001, 0.006) | 0.323 (0.296, 0.351)*** |
| Memory score (ordered recall) | Dietary diversity | | -0.003 (-0.066, 0.059) | | -0.002 (-0.011, 0.006) | -0.006 (-0.070, 0.059) |
|  | Hemoglobin concentration | | 0.071 (0.032, 0.110)** | | 0.009 (0.001, 0.017) | 0.080 (0.040, 0.120)** |
|  | Length-for-age z-score | | 0.056 (-0.006, 0.118) | | 0.037 (0.018, 0.057)** | 0.093 (0.031, 0.156)* |
|  | Motor development score | | 0.039 (0.023, 0.055)*** | | -0.001 (-0.003, 0.001) | 0.038 (0.021, 0.054)*** |
| Memory score (target actions) | Dietary diversity | | 0.003 (-0.097, 0.103) | | 0.001 (-0.014, 0.016) | 0.004 (-0.099, 0.107) |
|  | Hemoglobin concentration | | 0.067 (-0.009, 0.144) | | 0.020 (0.004, 0.036)* | 0.087 (0.007, 0.168) |
|  | Length-for-age z-score | | 0.054 (-0.043, 0.150) | | 0.089 (0.054, 0.124)*** | 0.143 (0.042, 0.243)* |
|  | Motor development score | | 0.083 (0.058, 0.109)*** | | 0 | 0.083 (0.058, 0.109)*** |
| Executive function (Overcome perseverative error) | Dietary diversity | | 0.031 (-0.029, 0.092) | | 0.001 (-0.004, 0.006) | 0.032 (-0.030, 0.093) |
|  | Hemoglobin concentration | | 0.064 (0.000, 0.117) | | 0.006 (0.000, 0.011) | 0.069 (0.017, 0.121)* |
|  | Length-for-age z-score | | 0.014 (-0.057, 0.085) | | 0.022 (-0.001, 0.045) | 0.036 (-0.042, 0.114) |
|  | Motor development score | | 0.017 (-0.002, 0.036) | | 0.001 (-0.001, 0.003) | 0.018 (-0.001, 0.038) |
| Executive function (Any tolerated delay) | Dietary diversity | | 0.029 (-0.037, 0.095) | | 0 | 0.029 (-0.037, 0.095) |
|  | Hemoglobin concentration | | 0.050 (-0.003, 0.102) | | 0.005 (0.000, 0.010) | 0.054 (0.002, 0.107) |
|  | Length-for-age z-score | | 0.033 (-0.032, 0.098) | | 0.016 (-0.004, 0.036) | 0.049 (-0.017, 0.114) |
|  | Motor development score | | 0.014 (-0.003, 0.032) | | 0 | 0.015 (-0.003, 0.032) |
| ^1^Values are β coefficients (95% CI). All adjusted for clustering at the health sub-center level. Adjusted analyses accounting for age of child in months, child sex, intervention group, wealth quintile of the household, religion, caste, and maternal education. Estimates of zero are not shown. * P<0.05; ** P<0.01; *** P<0.001. | | | | | | |

Supplemental Table 3: R^2^ values for path model

| **Dependent variable** | **R^2^** |
| --- | --- |
| Dietary diversity | 0.011 |
| Hemoglobin concentration | 0.037 |
| Length-for-age z-score | 0.081 |
| Motor development score | 0.312 |
| Stimulation score | 0.100 |
| Language development score | 0.373 |
| Personal-social development score | 0.360 |
| Memory score (pairs of actions) | 0.122 |
| Memory score (number of actions) | 0.111 |
| Executive function score (tolerated delay) | 0.050 |
| Perseverative error | 0.060 |
